# A *var* Gene Upstream Element Controls Protein Synthesis at the Level of Translation Initiation in *Plasmodium falciparum*

**DOI:** 10.1371/journal.pone.0100183

**Published:** 2014-06-17

**Authors:** Nicolas M. B. Brancucci, Kathrin Witmer, Christoph Schmid, Till S. Voss

**Affiliations:** 1 Department of Medical Parasitology and Infection Biology, Swiss Tropical and Public Health Institute, Basel, Switzerland; 2 University of Basel, Basel, Switzerland; Bernhard Nocht Institute for Tropical Medicine, Germany

## Abstract

Clonally variant protein expression in the malaria parasite *Plasmodium falciparum* generates phenotypic variability and allows isogenic populations to adapt to environmental changes encountered during blood stage infection. The underlying regulatory mechanisms are best studied for the major virulence factor *P. falciparum* erythrocyte membrane protein 1 (PfEMP1). PfEMP1 is encoded by the multicopy *var* gene family and only a single variant is expressed in individual parasites, a concept known as mutual exclusion or singular gene choice. *var* gene activation occurs *in situ* and is achieved through the escape of one locus from epigenetic silencing. Singular gene choice is controlled at the level of transcription initiation and *var* 5′ upstream (*ups)* sequences harbour regulatory information essential for mutually exclusive transcription as well as for the trans-generational inheritance of the *var* activity profile. An additional level of control has recently been identified for the *var2csa* gene, where an mRNA element in the 5′ untranslated region (5′ UTR) is involved in the reversible inhibition of translation of *var2csa* transcripts. Here, we extend the knowledge on post-transcriptional *var* gene regulation to the common *upsC* type. We identified a 5′ UTR sequence that inhibits translation of *upsC*-derived mRNAs. Importantly, this 5′ UTR element efficiently inhibits translation even in the context of a heterologous upstream region. Further, we found *var* 5′ UTRs to be significantly enriched in uAUGs which are known to impair the efficiency of protein translation in other eukaryotes. Our findings suggest that regulation at the post-transcriptional level is a common feature in the control of PfEMP1 expression in *P. falciparum*.

## Introduction

During intra-erythrocytic development, the human malaria parasite *Plasmodium falciparum* exports the major virulence factor erythrocyte membrane protein 1 (PfEMP1) to the red blood cell (RBC) surface [1]. The highly polymorphic N-terminal portion of PfEMP1 interacts specifically with a diverse set of endothelial host cell receptors such as CD36, ICAM1 or CSA [2], [3]. Due to the adhesive properties of this integral membrane component, infected RBCs (iRBCs) disappear from peripheral circulation and thus avoid clearance in the spleen. The resulting aggregation of infected erythrocytes within the microvasculature of various organs is linked to severe forms of the disease such as cerebral or placental malaria [4].

In order to escape humoral immune responses *P. falciparum* employs antigenic variation of PfEMP1. The underlying mechanisms are based on a complex interplay of transcriptional and epigenetic control processes [5]. PfEMP1 is encoded by the multicopy *var* gene family, the members of which predominantly locate within subtelomeric domains [6]–[9]. In addition, some *var* genes occur in tandem clusters in central areas of some chromosomes. Frequent recombination events generated a virtually limitless PfEMP1 sequence diversity that reflects the selective pressure acting on this immune-dominant antigen [10]–[12]. Notably, of the 60 *var* genes encoded in the haploid parasite genome, only a single variant is active at any given time [13]. This singular *var* gene choice is regulated at the level of RNA polymerase II-mediated transcription initiation and results in mutually exclusive expression of PfEMP1 [14]. Each *var* gene represents a fully functional genomic unit that is associated with either of four conserved 5′ upstream (*ups*) regions (*upsA, B, C* and *E*) [15]. *var* promoters are equipped with *cis*-acting elements to control transcriptional activation and repression and the inclusion of each gene into the programme of singular *var* activity [16]–[20]. Several studies identified a central contribution of epigenetic mechanisms to the control of *var* gene transcription. Subtelomeric and chromosome-internal *var* genes reside within heterochromatic, transcriptionally inert domains that cluster at the nuclear periphery [12], [21]–[25]. The silenced and active states of *var* genes are earmarked by the differential occurrence of specific post-translational histone modifications. Most prominently, transcriptionally silenced *var* loci are associated with nucleosomes that harbour histone 3 tri-methylated at lysine 9 (H3K9me3) and heterochromatin protein 1 (HP1) [24], [26]–[28]. HP1 binds to H3K9me3 and represents a major component of transcriptionally silent chromatin in eukaryotes [29]. The process of *var* gene activation occurs *in situ* and is accompanied by nuclear re-positioning of a formerly silenced locus into a transcriptionally competent perinuclear compartment [13], [18], [30], [31]. In contrast to silenced loci, the active *var* gene is associated with H3K9 acetylation and H3K4me2/3 as well as with the histone variants H2A.Z and H2B.Z in the *ups* region [26], [32]. While in most of all cases daughter cells recapitulate the *var* transcription pattern of their progenitors due to epigenetic inheritance, occasional switching events result in antigenic variation of PfEMP1 [33], [34]. In line with the essential roles of histone modifying enzymes in this process, recent studies observed the partial or complete breakdown of singular *var* gene choice in response to interfering with histone de-acetylation [30], [35] or H3K36 methylation [36].

Generally, the molecular mechanisms regulating gene expression in *P. falciparum* are only poorly understood. Transcriptome profiling studies revealed that most genes, including the *var*s, exhibit a specific temporal activity pattern during the 48 hour intra-erythrocytic developmental cycle (IDC), suggesting that malaria parasites use gene-specific transcriptional activation and repression to produce transcripts only when their gene products are required [37]–[40]. However, in spite of similarities between the wave-like transcript and protein abundance profiles, crude mRNA and protein levels are only rarely in direct correlation [41]–[44], indicating that post-transcriptional mechanisms significantly contribute to the control of protein expression in *P. falciparum*. More specifically, according to mathematical models, the rates of mRNA translation and protein degradation account for most of the observed discrepancies [41].

In other life cycle stages, parasites make use of diverse strategies to store and re-access pre-synthesised transcripts. The release of mRNA from translational repression in gametocytes and salivary gland sporozoites allows for a fast adaptation upon the change of host. Prior to gametocyte transmission, transcripts essential for ookinete formation are repressed and stabilised with the help of DOZI, a conserved DEAD-box RNA helicase [45], [46]. At least for a subset these transcripts, translational repression is mediated by a conserved U-rich element found in either of the 5′ or 3′ untranslated region (UTR) [47]. Sporozoites employ a different mechanism to inhibit protein synthesis. Here, the phosphorylation of eukaryotic initiation factor 2α (eIF2α) by IK2, a serine/threonine protein kinase, results in a global suppression of translation and thus prevents cells in the salivary gland from pre-mature development into liver stage parasites [48]. Interestingly, the expression of a particular *var* gene, *var2csa*, is also under specific post-transcriptional control [49]–[51]. VAR2CSA mediates adherence of iRBCs to chondroitin sulphate A (CSA) on placental syncytiotrophoblasts, which is responsible for pregnancy-associated malaria [3], [52], [53]. *var2csa* expression is controlled by the unique *upsE* upstream sequence [9], [15], and translation of the *var2csa* mRNA is reversibly repressed by the presence of a 360 bp upstream open reading frame (uORF) [49]. This process is independent from expression of the uORF-encoded polypeptide and translational re-initiation was recently reported as the rate-limiting step of VAR2CSA synthesis [51]. Other documented evidence for the involvement of post-transcriptional mechanisms in the control of *var* genes is lacking.

We recently identified a 101 bp target sequence (MEE) in the upstream region of an *upsC var* gene that is essential for singular *var* gene choice [54]. Here, we show that in addition to its role as a *cis*-acting DNA sequence, the MEE element acts on the level of the mRNA by inhibiting translation of *upsC*-derived transcripts. Our data suggest that post-transcriptional regulation of *var* gene expression may be a common mechanism in the control of mutually exclusive expression of PfEMP1.

## Results

### A *var* Gene Upstream Element Inhibits Heterologous Promoter Activity

**Figure 1:**
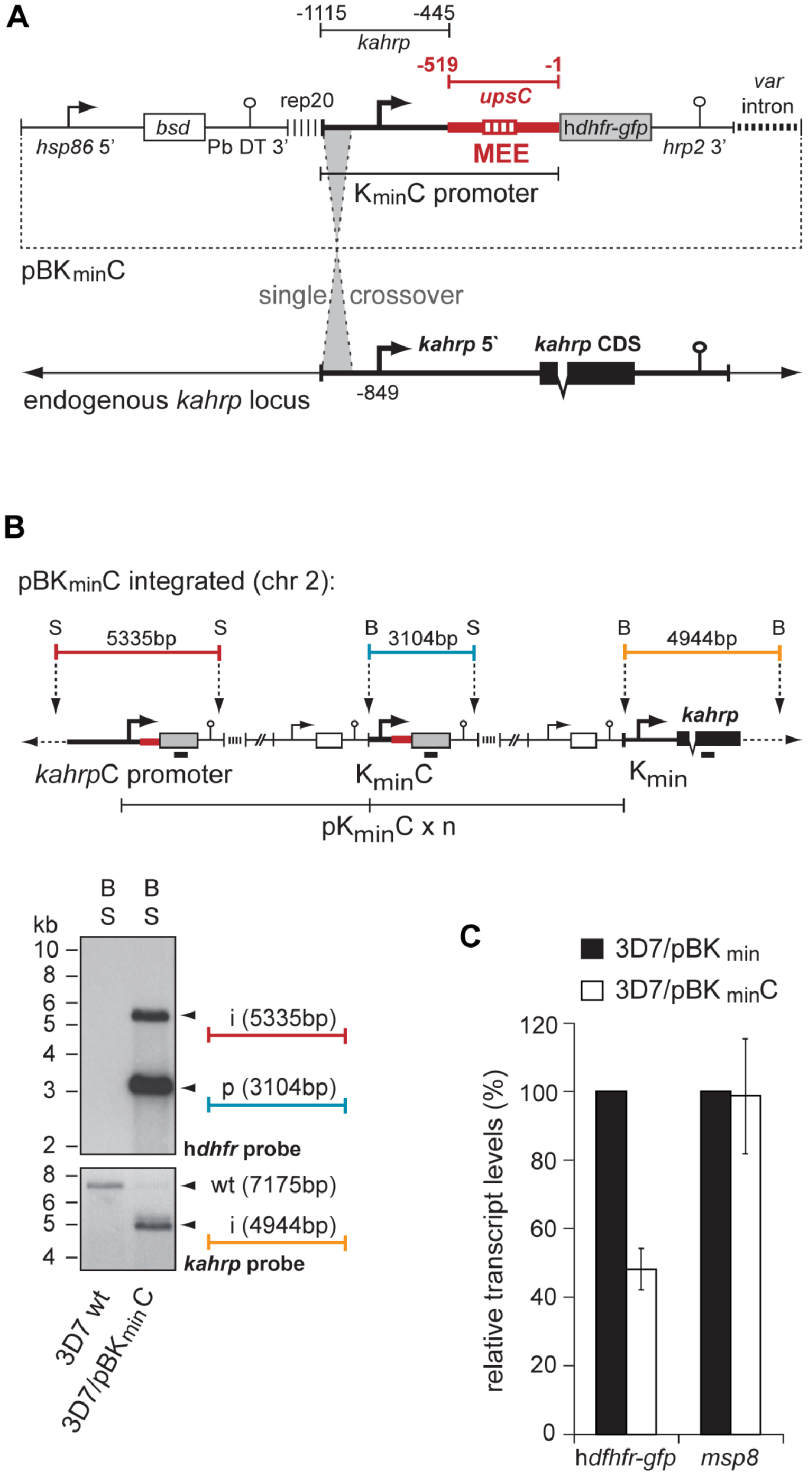
Integration of the *upsC* 5′ upstream sequence into a heterologous context at the *kahrp* locus. (A) Schematic map of the transfection construct pBK_min_C. Single-crossover integration was guided by *kahrp* 5′ homology. The position of the *kahrp* TSS is indicated [81]. Numbers refer to the nucleotide positions relative to the ATG start codon. The *bsd* resistance cassette selects for stably transfected parasites. The *var* intron is indicated by a bold dashed line. hsp86 5′, *hsp86* promoter; Pb DT 3′, *P. berghei dhfr*-thymidylate synthase terminator; rep20, 0.5 kb TARE6 repeat element; hrp2 3′; histidine-rich protein 2 terminator. MEE, location of the 101 bp mutual exclusion element MEE [54]. (B) Genomic situation after integration of the pBK_min_C concatamer into the endogenous *kahrp* locus. Restriction sites used in Southern analysis and fragment lengths are indicated and colour-coded. S, *Stu*I; B, *Bgl*II. The Southern blot on *Bgl*II/*Stu*I-digested gDNA shows integration of pBK_min_C into the endogenous locus of *kahrp*. The membrane was hybridised with h*dhfr* (top) and *kahrp* (bottom). Fragments are colour-coded according to the integration map. wt, size of the *kahrp* fragment in 3D7 wild-type parasites. i, integration event; p, plasmid fragment. (C) The *upsC* 5′ UTR sequence represses *kahrp* promoter activity. The bars represent the ratio of relative h*dhfr-gfp* and *msp8* transcript levels in 3D7/pBK_min_C parasites (open bars) compared to the 3D7/pBK_min_ control (black bars) cultured in absence of WR. Results are the mean +/− s.d. of three independent experiments. Values are normalised for PF3D7_1331700 transcripts.

The 101 bp MEE element is located downstream of the transcriptional start site (TSS) in the *upsC* upstream region and controls inclusion of the locus into the programme of mutually exclusive *var* activity [54]. Here, we aimed at a more detailed functional characterisation of this regulatory sequence. First, we asked whether an *upsC* upstream sequence including the MEE is able to modulate gene expression autonomously when placed in a conserved position downstream of the TSS of a heterologous promoter. To achieve this, we used our previously published transfection vector pBK_min_ as a vehicle to target the endogenous *kahrp* (knob-associated histidine rich protein) locus [54]. pBK_min_ contains the blasticidin deaminase (*bsd*) resistance gene followed by a reporter cassette in which a minimal *kahrp* promoter (K_min_) controls expression of the h*dhfr*-*gfp* (human dihydrofolate reductase fused to green fluorescent protein) reporter gene that confers resistance to the antifolate WR99210 (WR). Here, we replaced the region spanning bps −445 to −1 downstream of the TSS of the minimal *kahrp* promoter with the *upsC* sequence (bps −519 to −1) containing the MEE (Figure 1A). Transfected 3D7 parasites were selected on blasticidin-S-HCl (BSD) and the plasmid was integrated into the endogenous *kahrp* locus by single-crossover homologous recombination (3D7/pBK_min_C). This event created the *kahrp*-*upsC* hybrid upstream sequence *kahrpC* that drives expression of the h*dhfr*-*gfp* gene (Figure 1B). In this context, the wild-type *kahrp* promoter drives transcription of h*dhfr-gfp* and produces transcripts in which the 5′ UTR of *kahrp* had been swapped with that of *var upsC*. Each of the downstream reporter cassettes on the integrated concatamer is flanked by the minimal K_min_C 5′ upstream region, whereas the endogenous *kahrp* gene is controlled by the minimal K_min_ sequence. Note that these units are essentially inactive because K_min_ has negligible promoter activity [54].

Surprisingly, 3D7/pBK_min_C parasites were completely refractory to WR selection in numerous independent challenge experiments. To test if this was due to a block in transcription we performed quantitative reverse transcription-PCR (qRT-PCR) analysis. 3D7/pBK_min_C parasites consistently displayed two-fold lower h*dhfr-gfp* transcript levels (52.04% +/− 17.3 s.d.) compared to the control line 3D7/pBK_min_ [54] where the wild-type *kahrp* upstream sequence controls h*dhfr-gfp* transcription (Figure 1C). While this result indicated that the *upsC* upstream sequence has a negative impact on *kahrp* promoter activity, a two-fold reduction in steady state transcript levels alone was unlikely to account for the irrevocable sensitivity of 3D7/pBK_min_C to WR. Sequencing of reporter transcripts further excluded a scenario in which deleterious mutations may have been responsible for this prominent phenotype (data not shown).

### The *var* Gene 5′ UTR Inhibits Translation of h*dhfr-gfp* Reporter Transcripts

**Figure 2:**
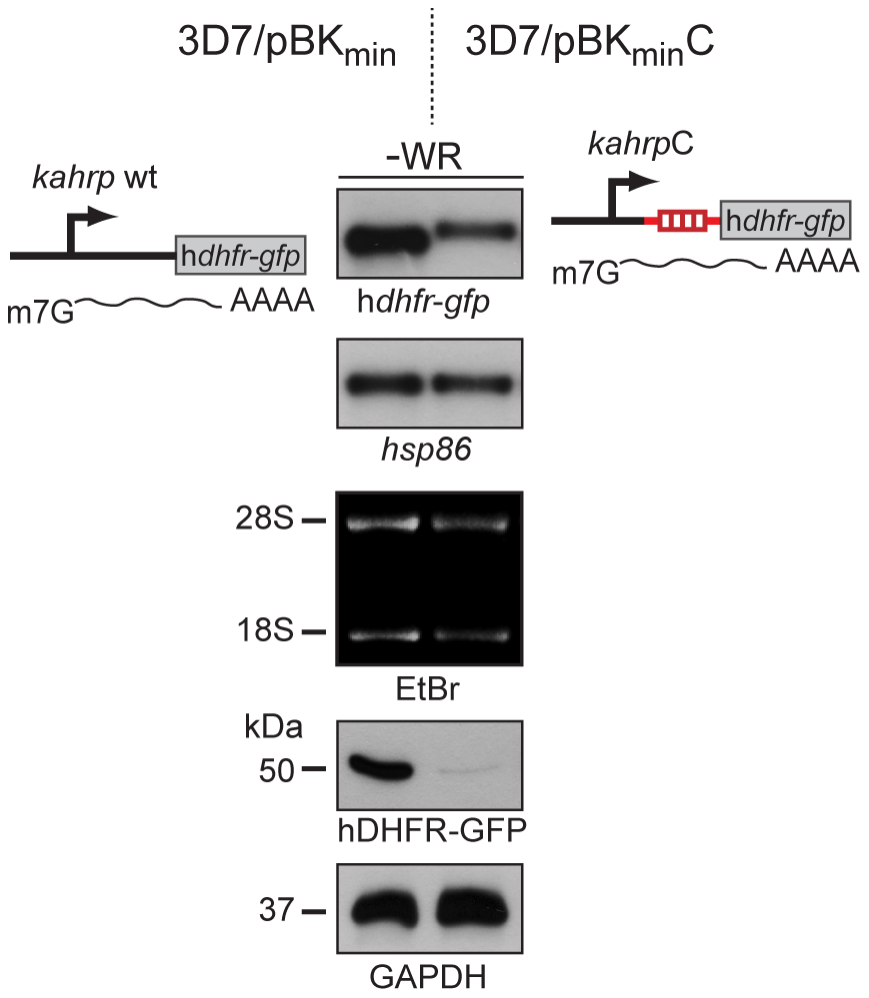
The *upsC* 5′ UTR element inhibits translation. Semi-quantitative analysis of transcript and protein abundance in 3D7/pBK_min_ (control) and 3D7/pBK_min_C ring stage parasites (6–14 hpi) cultured in absence of WR (−WR). Top panels: h*dhfr-gfp* and *hsp86* (loading control) transcripts were detected by Northern blot. Ethidium bromide-stained 18S and 28S rRNAs serve as second loading control. Bottom panels: expression of hDHFR-GFP and GAPDH (loading control) in the same parasite samples were analysed by Western blot.

In order to assess possible effects of the *upsC* 5′ UTR on the post-transcriptional level we performed parallel semi-quantitative Northern and Western blot analyses (Figure 2). These experiments confirmed the reduced h*dhfr-gfp* transcript levels in 3D7/pBK_min_C compared to the control line 3D7/pBK_min_, which we already observed by qRT-PCR (Figure 1C). As expected, *kahrpC*-derived transcripts had a slightly increased size compared to those originating from the wild-type promoter, demonstrating that the h*dhfr-gfp* mRNA was correctly transcribed in 3D7/pBK_min_C. Strikingly, however, despite the presence of substantial amounts of steady state h*dhfr-gfp* transcripts, 3D7/pBK_min_C parasites hardly expressed the hDHFR-GFP protein (Figure 2). This result provides direct evidence for an important function of the *upsC* 5′ UTR in translational inhibition and explains the refractoriness of 3D7/pBK_min_C parasites to WR selection.

**Figure 3:**
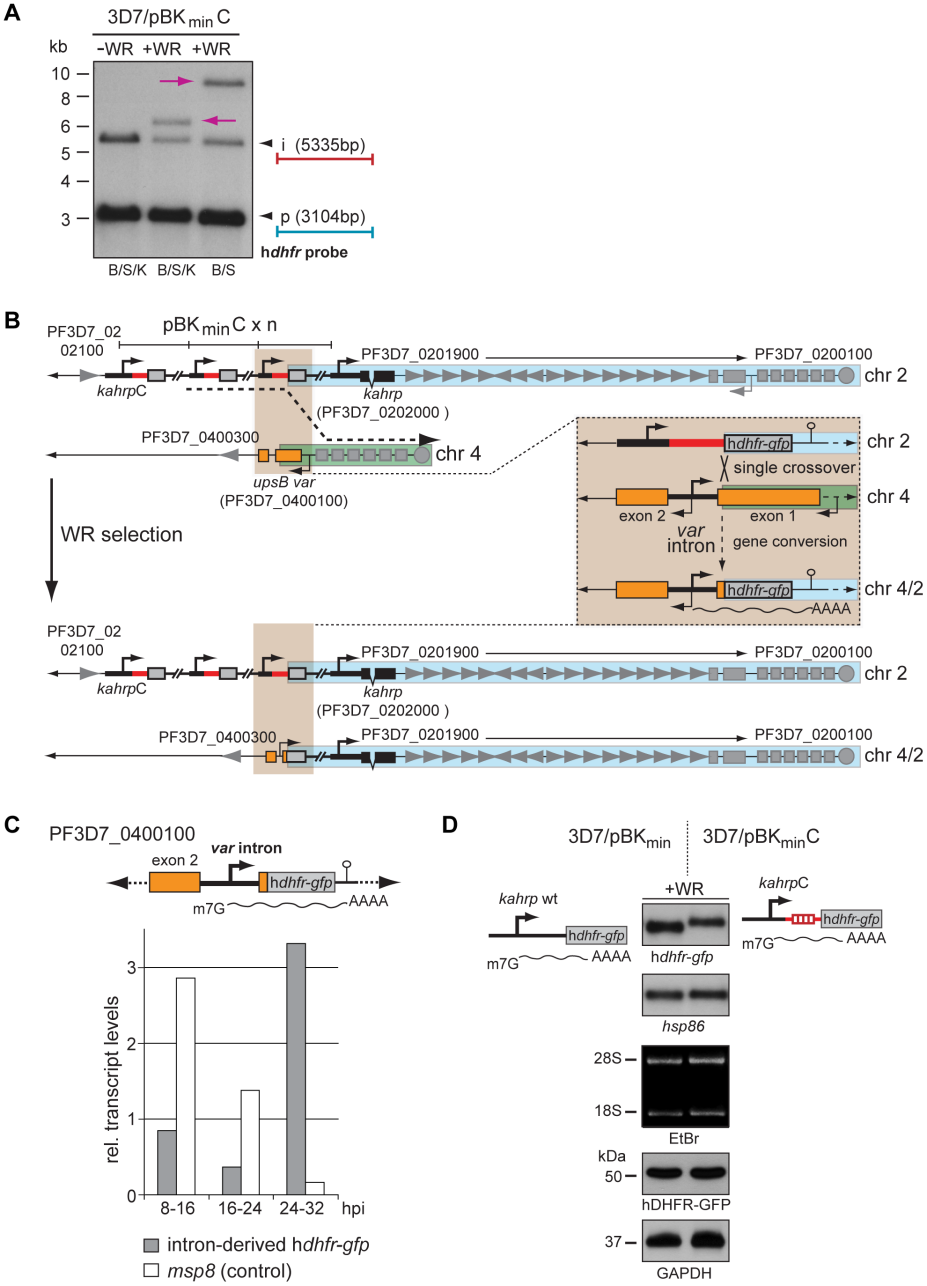
A gene conversion event revokes translational inhibition of h*dhfr-gfp* transcripts. (A) Southern analysis on digested gDNA from unselected and WR-selected 3D7/pBK_min_C parasites. Additional h*dhfr*-containing fragments detected in WR-selected parasites only are highlighted by pink arrows. S, *Stu*I; B, *Bgl*II; K, *Kpn*I; i, integration event; p, plasmid fragment. (B) The ends of chromosome 2 and 4 in unselected and 4/2 in WR-selected parasites are schematically depicted. Gene IDs (www.plasmoDB.org) are indicated for a subset of genes as reference. The dashed arrow highlights the site of gene conversion. The blue box represents the duplicated region of chromosome 2. The green box represents the region of chromosome 4 that was deleted. The brown box displays a zoom-in view of the gene conversion event and the resulting recombined locus. Detailed mapping and identification of the recombination site is presented in Figures S1 and S2. (C) h*dhfr-gfp* transcripts are produced from the *var* gene intron on chromosome 4 in WR-selected 3D7/pBK_min_C parasites. Values represent relative *var* intron-derived h*dhfr-gfp* (grey bars) and ring stage-specific *msp8* (open bars, control) transcript levels at three consecutive time points in WR-selected 3D7/pBK_min_C parasites (normalised to PF3D7_1331700 transcripts). hpi, hours post invasion. (D) Semi-quantitative analysis of transcript and protein abundance in 3D7/pBK_min_ (control) and 3D7/pBK_min_C ring stage parasites (6–14 hpi) cultured in presence of WR99210 (+WR). Top panels: h*dhfr-gfp* and *hsp86* (loading control) transcripts were detected by Northern blot. Ethidium bromide-stained 18S and 28S rRNAs serve as second loading control. Bottom panels: expression of hDHFR-GFP and GAPDH (loading control) in the same parasite samples were analysed by Western blot.

Noteworthy, after twelve unsuccessful drug challenges we were eventually able to select for a WR-resistant 3D7/pBK_min_C population. In light of these difficulties in generating a WR-resistant line, we considered a genomic rearrangement the most plausible cause for this altered phenotype. Indeed, Southern blot analysis revealed an additional h*dhfr-gfp* fragment in WR-selected compared to unselected parasites (Figure 3A). To determine this recombination event in exact detail, we carried out an elaborate mapping strategy based on further Southern blotting, ligation-mediated PCR and DNA sequencing (Figures S1 and S2). These efforts uncovered a major gene conversion event that resulted in the exchange of the end of chromosome 4 with a duplicated version of the end of chromosome 2 (Figure 3B). This occurred through the homologous recombination between a 10 bp sequence directly upstream of the most telomere-proximal h*dhfr-gfp* gene on chromosome two and an identical 10 bp sequence at the exon 1-intron boundary of *var* gene PF3D7_0400100 (Figures S1 and S2). Consequently, transcription of a single h*dhfr-gfp* gene in WR-resistant 3D7/pBK_min_C parasites was now under control of the reverse strand of a *var* gene intron. Notably, the *var* intron possesses bi-directional promoter activity [55], [56]. Indeed, qRT-PCR using primers specific to this recombined locus unambiguously identified active *var* intron-driven h*dhfr-gfp* transcription in WR-selected 3D7/pBK_min_C parasites (Figure 3C), which resulted in successful expression of hDHFR-GFP (Figure 3D). In line with the peak of intron promoter activity late during the IDC [55], synthesis of intron-derived h*dhfr-gfp* transcripts was higher in trophozoites/early schizonts compared to ring stages (Figure 3C). Attempts to identify intron-derived mRNA by Northern blotting were unsuccessful (data not shown). We explain this by the low abundance of intron-derived transcripts and similar expected size compared to those originating from the *kahrpC* promoter. Furthermore, intron-mediated antisense transcription initiates at variable sites [55], which additionally hampers detectability in Northern analysis.

The emergence of intron-derived h*dhfr-gfp* mRNA in WR-selected cells confirms that the lack of hDHFR-GFP expression in unselected 3D7/pBK_min_C parasites is solely caused by translational inhibition of *kahrpC*-derived transcripts through the *upsC* 5′ UTR. Importantly, the fact that bypassing this restriction was only possible through an extremely rare recombination event underscores the efficiency at which the *upsC* 5′ UTR inhibits translation. In summary, we conclude that the *upsC* 5′ upstream sequence investigated here exhibits a dual role in regulating expression; (i) as a DNA element it has a repressive effect on RNA PolII-dependent transcription, and (ii) as a 5′ UTR element it efficiently prevents translation.

### The MEE Inhibits Translation of *var* Transcripts

**Figure 4:**
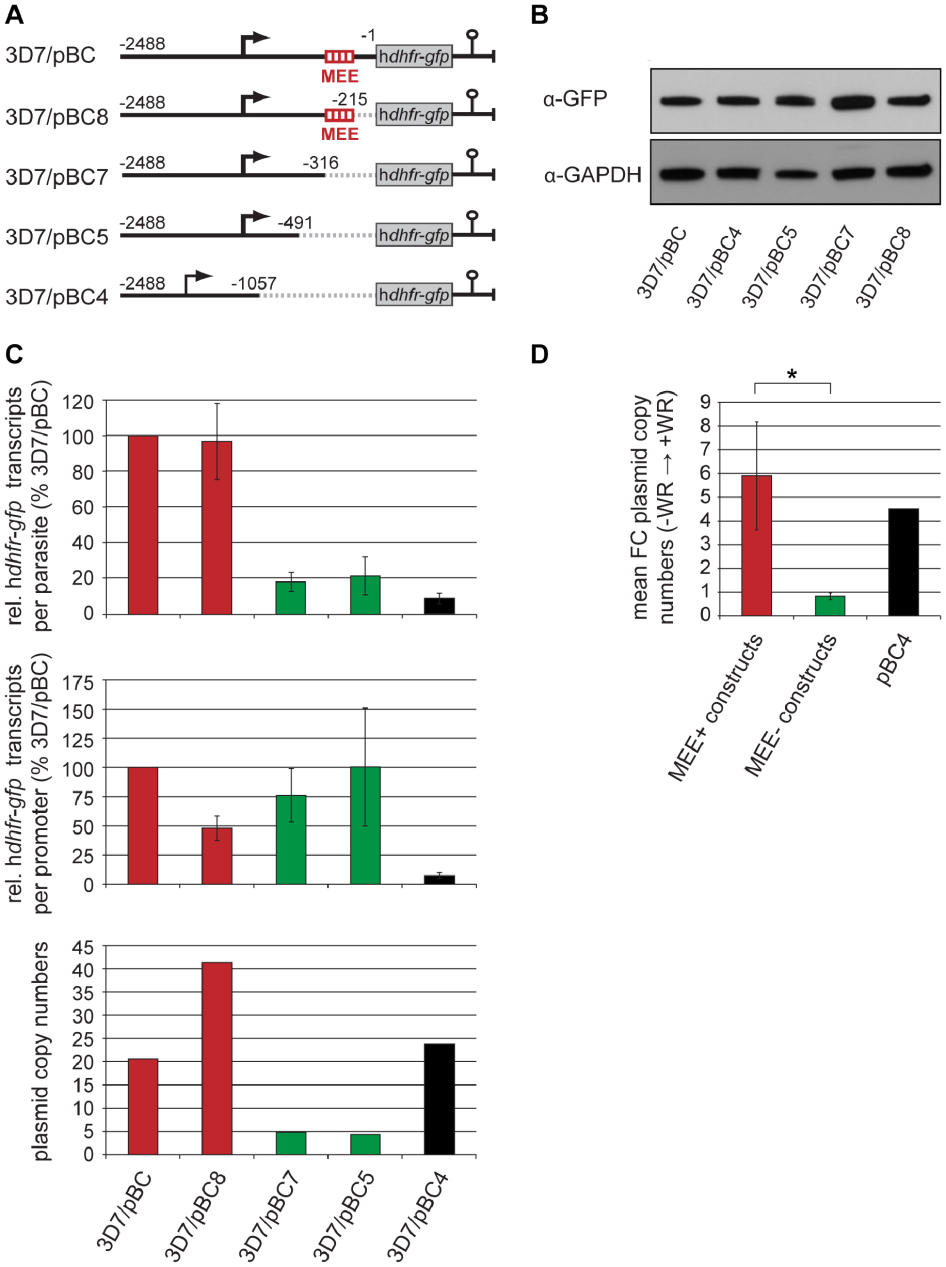
The MEE inhibits translation in the natural context of the *upsC* promoter. (A) Schematic depiction of *upsC var* promoter reporter constructs [54]. Deletions are represented by dashed lines. Numbers refer to the nucleotide positions relative to the ATG start codon. The position of the MEE is highlighted. (B) Expression of hDHFR-GFP and GAPDH (loading control) in WR-selected parasites was analysed by semi-quantitative Western blot. (C) Top panel: Proportion of total steady-state h*dhfr-gfp* transcripts in WR-selected parasites carrying truncated upstream sequences relative to the control line 3D7/pBC. Values are derived from three independent experiments (mean +/− s.d.) (normalised to PF3D7_1331700 transcripts). Middle panel: Proportion of steady-state h*dhfr-gfp* transcripts produced by a single promoter in WR-selected parasites carrying truncated upstream sequences relative to the control line 3D7/pBC. Values represent the data displayed in the top panel divided by the average plasmid copy number determined from the same batch of parasites (bottom panel). (D) Mean increase in plasmid copy numbers (+/− s.d.) after WR selection in parasites transfected with constructs carrying MEE-positive upstream sequences (red) or MEE-negative upstream sequences (green). The increase in plasmid copy numbers in WR-selected 3D7/pBC4 is shown in black. Individual plasmid copy numbers determined for each population are shown in Figure S3. Asterisk, p = 0.0015 (Student’s t-test).

In light of the above findings, we reasoned that deletion/truncation of the corresponding 5′ UTR sequence from the context of the *upsC* upstream region will lead to enhanced translation. To confirm this hypothesis we used a previously established set of WR-selected parasite lines carrying episomal plasmids [54] to investigate the effect of *upsC* 5′ UTR truncations on steady state h*dhfr-gfp* transcript levels and hDHFR-GFP expression using parallel qRT-PCR and semi-quantitative Western blot analyses (Figure 4). In pBC the “full length” 2.5 kb *upsC* upstream sequence (−2488 to −1 with respect to the ATG start codon) controls transcription of the h*dhfr-gfp* reporter. In pBC8, pBC7, pBC5 and pBC4 deletions of increasing length have been introduced directly upstream of the ATG (Figure 4A). Note that none of these truncations alters the temporal activity profile of the *upsC* promoter, but the 1057 bp deletion in pBC4 affects the TSS and transcription initiates from a weak alternative upstream TSS [54]. As shown in Figure 4B all WR-selected cell lines expressed similar amounts of hDHFR-GFP protein. However, 3D7/pBC and 3D7/pBC8 parasites displayed five- to over ten-fold higher total h*dhfr-gfp* transcript levels per parasite compared to 3D7/pBC7, 3D7/pBC5 and 3D7/pBC4 (Figure 4C, top panel). This shows that both pBC- and pBC8-derived transcripts are indeed translated with substantially lower efficiency than those produced from pBC7, pBC5 and pBC4. Interestingly, these latter three constructs all lack the MEE element (−316 to −215 with respect to the ATG start codon [54]) (Figure 4A) suggesting the inhibitory effect may be mediated by this region.

When interpreting these results, it is important to consider the fact that episomal plasmids in *P. falciparum* exist as concatamers of tandemly repeated units [57]. Hence, unlike in 3D7/pBK_min_C parasites where a single promoter is responsible for the production of all h*dhfr-gfp* transcripts, the total h*dhfr-gfp* transcript levels in the 3D7/pBC series reflect the sum of transcripts produced simultaneously by multiple expression cassettes in each parasite. To account for this we determined the mean plasmid copy number per parasite in each transfected population and used these values to calculate the h*dhfr-gfp* transcript levels generated by a single promoter. Consistent with previous findings [54], all *upsC* upstream regions comprising an intact promoter and TSS (pBC, pBC8, pBC7, pBC5) generated similar amounts of steady state transcripts per unit, suggesting that the 5′ UTR deletions had no major impact on mRNA stability (Figure 4C, middle panel). However, 3D7/pBC and 3D7/pBC8 parasites carried five- to ten-fold more plasmids per parasite compared to 3D7/pBC7 and 3D7/pBC5 (Figure 4C, bottom panel) and this entirely explains the high levels of total h*dhfr-gfp* transcripts observed in 3D7/pBC and 3D7/pBC8 (Figure 4C, top panel). Most importantly, the increased plasmid copy numbers in both 3D7/pBC and 3D7/pBC8 are a direct result of WR selection itself; prior to WR challenge these parasites contained similarly low plasmid copy numbers as 3D7/pBC7 and 3D7/pBC5 (Figures 4D and S3). This demonstrates that unlike in 3D7/pBC7 and 3D7/pBC5, the amount of h*dhfr-gfp* transcripts available in 3D7/pBC and 3D7/pBC8 parasites prior to WR challenge was insufficient to facilitate expression of hDHFR-GFP levels required to confer WR resistance. Hence, since the *upsC* promoter in all transfected lines produced similar levels of steady state h*dhfr-gfp* mRNA, the transcripts flanked by a MEE-positive 5′ UTR (pBC and pBC8) were translated with lower efficiency compared to those where this element was absent (pBC7 and pBC5). Notably, two additional *upsC* constructs retaining the MEE (pBC1 and pBC2 [54]) also increase in copy numbers upon WR selection to a similar extent as pBC and pBC8. In contrast, WR challenge did neither select for increased plasmid copy numbers in 3D7/pBC7 and 3D7/pBC5 nor in two additional lines where *upsC* constructs also lack the MEE (3D7/pBC6.2 and 3D7/pBC5.2), or a control line where the unrelated *mahrp1* promoter controls h*dhfr-gfp* transcription (3D7/pBM [54]) (Figures 4D and S3). This clearly shows that in order to acquire WR resistance parasites expressing MEE-positive *upsC* transcripts must compensate for their poor translation efficiency by augmenting total h*dhfr-gfp* transcript levels through increasing plasmid copy numbers. Our findings obtained with 3D7/pBC4 parasites further corroborate these results. The regulatory region in pBC4 (which lacks the MEE element) generated very low amounts of h*dhfr-gfp* transcripts, which is due to the low activity of the alternative upstream TSS employed by this promoter [54] (Figure 4C, middle panel). Similar to pBC and pBC8, WR selection of 3D7/pBC4 parasites led to a substantial increase in plasmid copy numbers showing that the low level of h*dhfr-gfp* transcripts in these parasites was insufficient to confer WR resistance (Figure 4C, bottom panel and Figure 4D). However, the crucial difference between 3D7/pBC4 compared to 3D7/pBC and 3D7/pBC8 is that, although WR challenge selects for parasites carrying high plasmid copy numbers in all three lines, 3D7/pBC4 parasites acquire WR resistance with over 10-fold lower total h*dhfr-gfp* steady state transcripts compared to 3D7/pBC and 3D7/pBC8 (Figure 4C, top panel).

## Discussion

Here we describe the identification of an autonomous *cis*-acting element implicated in post-transcriptional *var* gene regulation. First, insertion of bps −519 to −1 of the *upsC* 5′ UTR into the context of the endogenous *kahrp* promoter rendered the corresponding hybrid transcripts incompetent for efficient translation. Second, the independent analysis of several truncated *upsC* sequences consistently showed that transcripts carrying a deletion of the 5′ UTR MEE element (nucleotides −316 to −215) gave rise to significantly higher hDHFR-GFP protein levels compared to transcripts carrying this region. These combined results demonstrate that the *upsC* 5′ UTR, or more precisely the MEE element, has a function in reducing the efficiency of translation.

Since hDHFR expression is subject to auto-regulation it is important to exclude the possibility that this mechanism may have been responsible for our observations. The hDHFR enzyme represses translation of its cognate mRNA by binding specifically to an 82 bp RNA element in the coding region [58]–[60]. In presence of substrates or inhibitors the enzyme dissociates from the mRNA, leading to a rapid release from translational inhibition and consequently increased hDHFR expression [58], [59], [61]. hDHFR auto-regulation occurs not only in human cells but also in *P. falciparum* transfected with h*dhfr*-encoding plasmids [62]. Zhang and Rathod reported that in presence of 500 nM WR a *P. falciparum* line expressing h*dhfr* displayed six-fold increased hDHFR expression at unchanged mRNA levels compared to the same parasites cultured in absence of drug [62]. The important difference between our and the above studies is that we did not compare hDHFR-GFP expression levels between identical cell lines cultured in presence or absence of inhibitor but rather between different parasites lines cultured under identical growth conditions. In this controlled setup, we observed that different h*dhfr-gfp* transcripts showed dramatically different capacities to support efficient translation. Parasites expressing h*dhfr-gfp* from the endogenous *kahrp* promoter expressed hDHFR-GFP and were easily selected on WR when transcripts were flanked by the wild type *kahrp* 5′ UTR. In striking contrast, when these transcripts were flanked by the *upsC* 5′ UTR parasites failed to translate functional levels of hDHFR-GFP and were completely refractory to WR selection. Since inhibitor-induced release of hDHFR auto-inhibition occurs rapidly within 24 hours after challenge [62] it is clear that the poor translation efficiency of these *kahrpC*-derived transcripts is not due to this mechanism but is mediated by the 519 bp *upsC* 5′ UTR instead. We obtained the same results with WR-selected parasite lines in which episomal *upsC* promoters drive h*dhfr-gfp* transcription. We consistently observed that h*dhfr-gfp* transcripts carrying a deletion of the corresponding 5′ UTR sequence were efficiently translated and these parasites readily acquired WR resistance with the pool of transcripts available prior to WR challenge. In contrast, transcripts retaining this sequence were inefficiently translated, which is entirely expected given that *kahrpC*- and *upsC*-derived transcripts are identical apart from the region upstream of position −519. However, unlike 3D7/pBK_min_C parasites, in which h*dhfr-gfp* transcription occurs from a single chromosomal locus, these populations were able to overcome WR sensitivity but this always required an increase in plasmid copy numbers (and consequently h*dhfr-gfp* transcript levels) by up to eight-fold compared to unselected parasites. Hence, even if the addition of 4 nM WR triggered partial or full release of hDHFR auto-inhibition in our cell lines (note that this concentration is 125-fold lower than that used by Zhang and Rathod [62]) this was clearly insufficient to relieve translational inhibition of transcripts flanked by the *upsC* 5′ UTR.

The process of translation can be divided into initiation, elongation and termination. Among these phases, protein synthesis in eukaryotes is most highly regulated during initiation [63], i.e. the rate at which ribosomes launch proper genesis of the peptide chain. Usually, initiation is characterised by the recruitment of the translation pre-initiation complex (PIC) to the m7G cap at the 5′ end of transcripts [64]. Once associated with mRNA, the PIC scans the untranslated region for downstream AUG start codons [65]. Both PIC recruitment and scanning can be impeded by secondary RNA structures, resulting in reduced initiation efficiency [66]. 5′ polarity of the scanning mechanism provides further means to regulate translation as the first encountered start codon usually serves as a unique site of initiation [65]. Because of this “first AUG rule”, upstream start codons (uAUGs) can interfere with translation, often through creating small upstream open reading frames (uORFs). The encoded peptides, however, are only rarely involved in translational inhibition. In the case of *P. falciparum var2csa*, initiation at an uORF indeed prevents translation from the regular start codon in a reversible manner and it has been suggested that this process may allow for rapid switching to the VAR2CSA PfEMP1 variant under favouring environmental conditions [49]. The mechanisms underlying *upsC*-mediated translational inhibition identified in this study appear to be distinct from those operating in *var2csa* regulation. This hypothesis is based on the observation that the inhibitory effect of the *upsC* 5′ UTR is irreversible, demonstrating that inefficient translation is a hard-wired feature of *upsC*-derived transcripts. This is evident from the fact that the poor translation efficiency of *upsC* 5′ UTR-containing transcripts could either not be reverted (in case of 3D7/pBK_min_C parasites) or had to be compensated for by increasing plasmid copy numbers and therefore h*dhfr-gfp* transcripts (in case of 3D7/pBC and related lines). Hence, in contrast to the uORF in *var2csa*, the *upsC* element is unlikely to be involved in adaptive processes but rather fulfils gene-intrinsic post-transcriptional regulatory functions.

**Figure 5:**
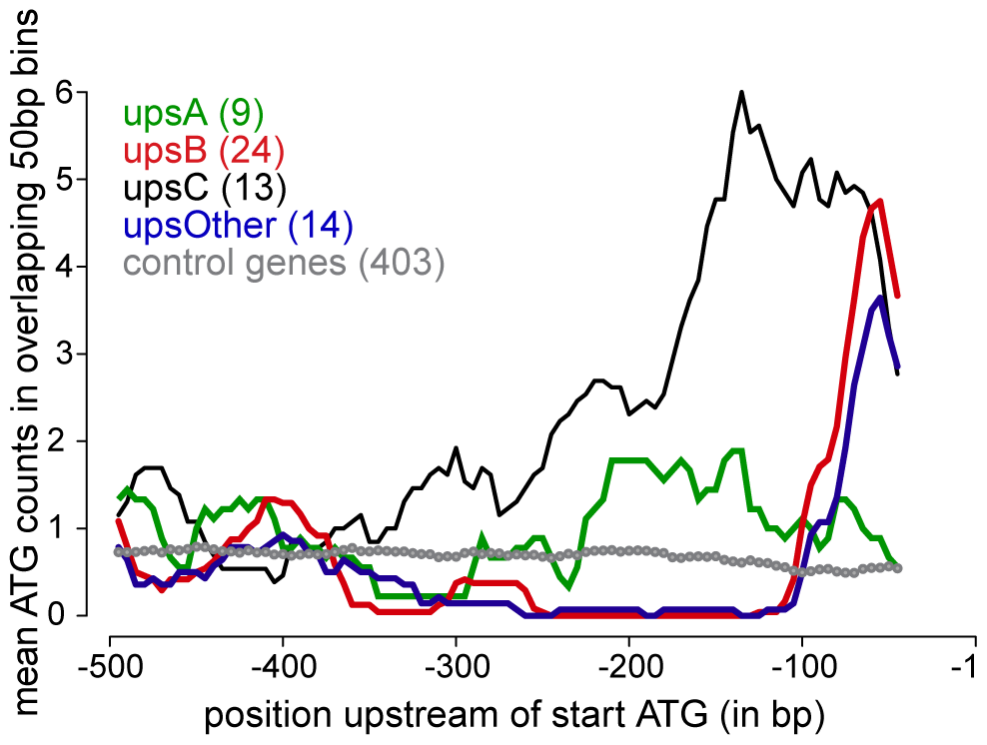
uAUGs are enriched in *var* 5′ UTRs. For each gene, sequences ranging from bp −500 to −1 relative to the ATG start codon were downloaded from PlasmoDB version 7.2 (www.plasmoDB.org) and the counts of the trinucleotide sequence ‘ATG’ were assessed in sliding windows of 50 bps using custom-made Perl scripts. The average ATG counts for each sequence set were plotted using the statistical analysis package R (www.r-project.org). The *var* gene set includes 60 sequences, subdivided into groups “*upsA*”, “*upsB*”, “*upsC*” and “others” (*upsB/C*, *upsB/A*, *upsE*) according to the classification by Lavstsen and colleagues [15]. The control set consists of 5′ UTR sequences of 403 genes with peak transcription in ring stages. Selection of these sequences was based on RNASeq data [39] according to the following criteria: timing of maximal expression: 8 hpi and 16 hpi; maximal expression ratio: 8-fold induction; maximum expression percentile: 30^th^ percentile. uAUGs are significantly enriched in *var* 5′ UTRs compared to the control set of ring stage-specific genes (p = 7.56×10^−11^; Welch t-test).

At this stage we do not know whether translation initiation at uAUGs and/or translation of uORFs is involved in the inhibitory function of the *upsC* 5′ UTR. It is plausible that translational inhibition is mediated in a uORF-independent fashion, for instance by secondary mRNA structures and/or sequence-specific RNA/protein interactions that may block PIC recruitment and/or scanning. Notably, however, we observed a prominent enrichment of uAUGs in *var* 5′ UTRs in general compared to other ring stage-specific transcripts (Figure 5). The investigated *upsC* sequence in pBK_min_C is no exception to that rule. In fact, the 519 bp 5′ UTR sequence contains the remarkable number of 33 uAUGs. Moreover, the 101 bp MEE sequence element alone carries six uAUGs that may serve as initiation sites for the translation of 6-11 amino acid (aa) peptides. If uORF-translation indeed plays a role in regulating expression of *var* genes other than *var2csa* remains to be investigated. Whereas the similar average size (4-6aa) of uORFs with a predicted function in yeast [67] supports such an assumption, conserved uORF-encoded peptides in *Drosophila* (70aa) [68] and the *var2csa* gene (120aa) [49] are much larger. Importantly, however, irrespective of whether translation is initiated within the *upsC* 5′ UTR or not, uAUGs can lead to a substantial decrease in translation efficiency and they were shown to have important roles in translational control during development and conditions of cell stress [69], [70]. Clearly, *P. falciparum* must have evolved mechanisms to bypass the “first AUG rule” in order to express PfEMP1. This may be achieved through the well-known mechanisms of leaky uAUG scanning, re-initiation after uORF translation (as demonstrated for VAR2CSA expression [51]), or by using cap-independent strategies to guide ribosomes directly to the regular start site [65]. Although the exact mechanism by which translation of *upsC*-derived mRNA is inhibited remains to be determined, our findings demonstrate that *P. falciparum* uses this type of control to modulate expression of PfEMP1 variants. Similar to our observations, the 5′ UTR of a *P. falciparum* house-keeping gene was recently identified to reduce translation efficiency [71], and a recent study based on polysome profiling suggests the regulation of translation by 5′ UTRs may be a widespread mechanism to control protein expression in *P. falciparum* [72].

What could be the function of this type of regulation in the control of *var* gene expression? The answer to this question may lie in limitations of epigenetic mechanisms to strictly control singular expression of *var* genes. *var* transcription occurs through the escape of a single family member from transcriptionally inert heterochromatin that is associated with *var* loci. This process is linked to locus repositioning and the removal of local repressive epigenetic marks within a specialised perinuclear zone dedicated to *var* gene transcription [18], [22], [23], [26]–[28], [30], [31], [36]. It has also been speculated that a unique *trans-acting* DNA-sequence, similar to the H-element-mediated activation of mammalian olfactory receptor genes [73], [74], may be involved in singular *var* gene choice [5]. However, recent efforts based on genome conformation capture technologies failed to detect such an element [75]. Irrespective of the exact mechanism underlying mutually exclusive *var* activation, silencing of all other *var* genes may not be entirely efficient. Indeed, there is evidence for the co-appearance of low levels of additional full-length *var* transcripts in individual parasites [76], [77]. Hence, the repressive effect of *var* 5′ UTRs may minimise the risk of translating low abundance *var* transcripts derived from incompletely silenced loci.

In summary, we show that the *upsC* 5′ UTR autonomously mediates efficient translational inhibition. Our data are indicative for an involvement of upstream AUGs in this process, potentially leading to uORF expression. While beyond any doubt transcriptional and epigenetic control mechanisms dominate mutually exclusive *var* gene control, the strength of the observed effect indicates that translational inhibition may significantly contribute to the control of PfEMP1 expression. In this context, it is interesting to note that both translational inhibition and mutually exclusive locus recognition are dependent on the MEE sequence element. It is therefore tempting to speculate that *P. falciparum* may have evolved a control strategy that utilises a single regulatory element to control *var* gene expression at both the transcriptional and the translational levels.

## Materials and Methods

### Parasite Culture and Transfection

*P. falciparum* 3D7 parasites were cultured as described previously [78]. Growth synchronisation was achieved by repeated sorbitol lysis [79]. Transfections were performed as described [18]. Parasites were selected on 2.5 µg/ml BSD-S-HCl and 4 nM WR99210. To obtain pBK_min_C, the K_min_ promoter in pBK_min_ [54] was replaced by a *Bgl*II/*Not*I-digested *kahrp* promoter fragment (−1115 to −445 bps) containing an additional *BamH*I restriction site at the 3′ end directly upstream of the *NotI* site. The resulting plasmid was digested with *BamH*I/*Not*I to insert the *upsC* 5′ UTR element (−519 to −1) of *var* gene PF3D7_1240600. Plasmids pBC5.2 and pBC6.2 were obtained by replacing the *var* upstream region in pBC with truncated *upsC* sequences using *Bgl*II and *Not*I. All other cell lines analysed in this paper have been described previously [54]. Primers are listed in Table S1.

### Western Blot Analysis

Detection of hDHFR-GFP and GAPDH (loading control) was performed on whole cell lysates of parasites harvested at 6–14 hpi. Primary antibody dilutions were: mouse anti-GFP (Roche Diagnostics, 11814460001), 1∶500; monoclonal mouse anti-GAPDH 1-10B [80], 1∶20,000.

### Quantitative Reverse Transcription PCR

Pre-synchronised parasites cultures were synchronised twice 16 hours apart to obtain an eight-hour growth window. Total RNA was isolated using Tri Reagent (Ambion) and further purified using the RNeasy Plus Mini Kit (Qiagen) for removal of gDNA. Residual gDNA was digested with TURBO DNA-free DNAse (Ambion). All samples were tested negative for contaminating gDNA by qPCR. RNA was reverse transcribed using the RETROscript Kit (Ambion). qPCR reactions for absolute transcript quantification of h*dhfr-gfp*, PF3D7_1331700 (glutamine-tRNA ligase), *msp8* and *var* intron-derived h*dhfr-gfp* were performed at final primer concentrations of 0.4 µM using SYBR Green Master Mix (Applied Biosystems) on a StepOnePlus Real-Time PCR System (Applied Biosystems) in a reaction volume of 12 µl. Plasmid copy numbers were determined by qPCR on gDNA isolated from the same parasite samples and calculated by dividing the absolute h*dhfr-gfp* copy numbers by the average value obtained for *msp8* or PF3D7_1331700. All reactions were run in duplicate yielding virtually identical Ct values. Serial dilutions of gDNA and plasmid DNA were used as standards for absolute quantification. Relative transcript values were calculated by normalisation against the house-keeping gene PF3D7_1331700. Primer sequences are listed in Table S1.

### Southern and Northern Blot Analysis

gDNA was digested with appropriate restriction enzymes overnight and separated on 0.5% TBE-buffered 0.7% agarose gels. Total RNA was isolated from saponin-released parasites using TriReagent (Ambion). RNA was glyoxylated for 1 h at 55°C in five volumes glyoxal reaction mixture and electrophoresis was performed using 1×BPTE-buffered 1.5% agarose gels. Blots were probed with ^32^P-dATP-labeled h*dhfr*, *kahrp* and *hsp86* PCR fragments (primers are listed in Table S1). Membranes were stripped by boiling in 0.1% SDS for 15 min in between hybridisations.

## Supporting Information

Figure S1 Confirmation of the gene conversion event by Southern blotting and ligation-mediated PCR. (A) The upper map schematically depicts the end of chromosome 2 including the integrated plasmid concatamer (blue box) in 3D7/pBK_min_C parasites. *kahrp* promoter sequences are depicted by thick black lines. The *upsC* 5′ UTR sequence is depicted in red. The grey circles and squares represent the telomeric tract and telomere-associated repeat elements (TAREs) 1–6, respectively. Arrowheads indicate ORFs. The gene accession number refers to the most telomere-proximal *upsB var* gene PF3D7_0200100. The lower map shows a zoom-in view of the integrated concatamer (blue box). Restriction sites used in Southern analysis are shown by vertical dashed arrows, and expected fragment lengths are indicated and colour-coded. The h*dhfr* probe used for hybridisation is shown below the h*dhfr-gfp* coding sequence (grey box). (B) The autoradiograph shows the hybridisation results obtained with the h*dhfr* probe after digesting 3D7/pBK_min_C gDNA from unselected (−WR) and selected (+WR) populations with *EcoR*V/*Nco*I (red), *EcoR*V/*Spe*I (blue) or *EcoR*V/*Stu*I (green). Note the presence of an additional h*dhfr*-containing fragment after each double-digest specifically in WR-selected, but not in unselected parasites (highlighted by purple arrows). In each case, the size of the additional fragment (schematically depicted to the bottom right) is approximately 2 kb smaller than the size of the *EcoR*V/*Nco*I, *EcoR*V/*Spe*I or *EcoR*V/*Stu*I plasmid fragments (depicted to the top right). This result suggested the presence of a novel *EcoR*V site upstream of a single copy of h*dhfr-gfp* (highlighted in purple). i, integration event; p, plasmid fragment. (C) Ligation-mediated PCR. gDNA from WR-selected 3D7/pBK_min_C parasites was digested with *EcoR*V and *Nco*I and ligated into *EcoR*V/*Nco*I-digested pET-41 (EMD Biosciences). To amplify *EcoR*V/*Nco*I restriction fragments containing the h*dhfr* coding sequence, a primary PCR reaction was performed using T7 and h*dhfr*_R1 (R1) as forward and reverse primers, respectively. The primary PCR product was diluted 1∶200 and used as template for a semi-nested PCR reaction using T7 and h*dhfr*_R2 (R2) as forward and reverse primers, respectively. This amplicon was then sequenced using primer h*dhfr*_R3 (R3). The nucleotide sequence is shown at the bottom (reversed sequence). It begins with an *EcoR*V site within the intron of *var* gene PF3D7_0400100 on chromosome 4 (orange letters) and continues into the 3′ end of exon 1 (purple box). The green letters highlight the 10 bp sequence involved in the recombination event between the *var* and h*dhfr-gfp* loci. The grey box represents the start of the h*dhfr-gfp* coding sequence. A detailed schematic view of the recombination event is depicted above the nucleotide sequence. A single-crossover occurred between the 10 bp sequence (green letters) directly upstream of the h*dhfr-gfp* reporter (grey box) on chromosome 2, and an identical sequence (green letters) at the very 3′ end of exon 1 of *var* gene PF3D7_0400100 (purple box) on chromosome 4. As a result, the h*dhfr-gfp* reporter (grey box) was placed under control of the *var* gene intron promoter (orange line) on the reverse strand via gene conversion.
(TIF)

Figure S2 Further verification of the gene conversion event between chromosomes 2 and 4 in WR-selected 3D7/pBK_min_C parasites. (A) The map schematically depicts the end of chromosome 2 including the integrated plasmid concatamer (blue box) in 3D7/pBK_min_C parasites. *kahrp* promoter sequences are depicted by thick black lines. The *upsC* 5′ UTR sequence is depicted in red. The grey circles and squares represent the telomeric tract and TAREs 1–6, respectively. Arrowheads indicate ORFs. The gene accession number refers to the most telomere-proximal *upsB var* gene PF3D7_0200100. The lower map shows a zoom-in view of the integrated concatamer (blue box). Restriction sites used in Southern analysis are shown by vertical dashed arrows, and expected fragment lengths are indicated and colour-coded. The h*dhfr* probe used for hybridisation is shown below the h*dhfr-gfp* coding sequence (grey box). *EcoR*I sites are absent from the plasmid sequence. Hence, the *EcoR*I sites up- and downstream of the integrated concatamer release a restriction fragment in the size of 6228 bps (chromosomal DNA) plus *n* times 9475 bps (entire plasmid length) according to the number of copies in the concatamer. (B) The map schematically depicts the end of wild-type chromosome 4 including *var* gene PF3D7_0400100 (orange box) in unselected 3D7/pBK_min_C parasites. The PF3D7_0400100 exon 1 probe used for hybridisation is shown below the coding sequence. The position of the *EcoR*I restriction site downstream of the *var* locus and the expected fragment length are indicated. (C) The map schematically depicts the end of chromosome 4 after the gene conversion event between chromosomes 2 and 4 in WR-selected 3D7/pBK_min_C parasites (“chromosome 4/2 end”). The border between the green and blue boxes identifies the site of single-crossover recombination. The green and blue boxes represent sequences of the acceptor (chromosome 4) and donor (chromosome 2), respectively, of the gene conversion event. Restriction sites used in Southern analysis are shown by vertical dashed arrows, and expected fragment lengths are indicated and colour-coded. (D) The autoradiograph shows the hybridisation results obtained after digesting 3D7/pBK_min_C gDNA from unselected (−WR) and selected (+WR) populations with *EcoR*I, *EcoR*I/*Nco*I or *EcoR*I/*Sac*II. The membrane was hybridised with h*dhfr* (top) and PF3D7_0400100 (bottom) probes. Arrows are colour-coded according to the integration maps shown in panels A-C and identify the expected restriction fragments. The red, orange and yellow arrows highlight the restriction fragments that contain the single h*dhfr-gfp* cassette driven by the *var* intron promoter on chromosome 4/2 specifically in WR-selected parasites. Hybridisation with the PF3D7_0400100 exon 1 probe highlights the terminal chromosome 4 *EcoR*I fragment in unselected 3D7/pBK_min_C parasites, which had been deleted from the genome in WR-selected parasites by the gene conversion event. i, integration event; p, plasmid fragment.
(TIF)

Figure S3 Plasmid copy numbers before and after WR selection. (A) Schematic depiction of *upsC* constructs that either retain the MEE (left panel; red) or lack the MEE (right panel; green) in the upstream sequence. Control plasmid pBM carries the *mahrp1* promoter that naturally lacks a MEE element. (B) Average plasmid copy numbers before WR selection (light colours) and after WR selection (dark colours) in parasites transfected with MEE-positive constructs (red) or MEE-negative constructs (green). Plasmid copy numbers have been determined by qPCR and were calculated by dividing the absolute h*dhfr-gfp* copy numbers by the values obtained for the single copy gene *msp8*.
(TIF)

Table S1 Primers used in this study.
(PDF)
