# Supplementary figures and images for: A var Gene Upstream Element Controls Protein Synthesis at the Level of Translation Initiation in Plasmodium falciparum
Source: PLoS One. 2014 Jun 17;9(6):e100183. doi: 10.1371/journal.pone.0100183 (PMC4061111; doi:10.1371/journal.pone.0100183)

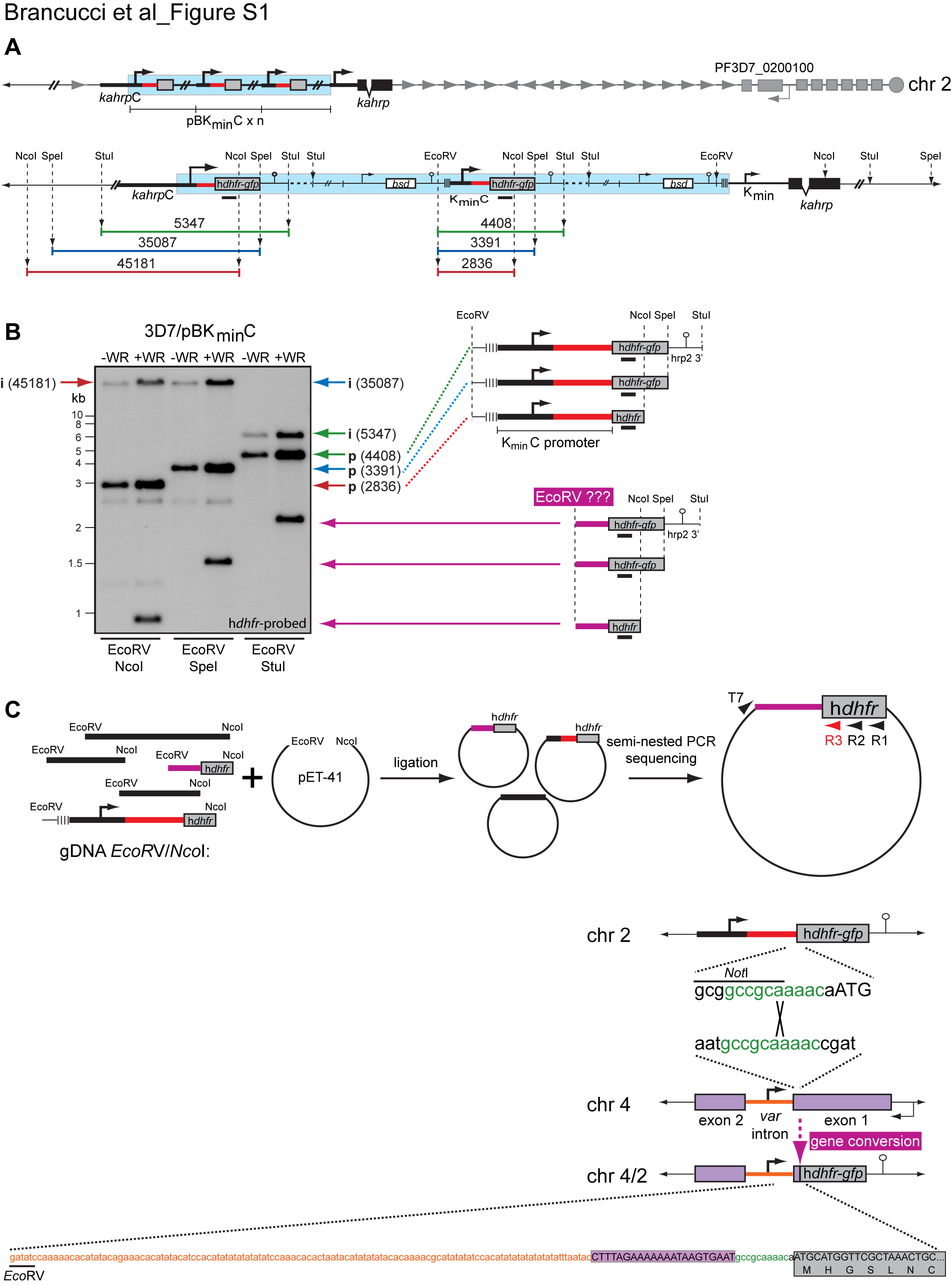

Supplement: Figure S1 — Confirmation of the gene conversion event by Southern blotting and ligation-mediated PCR. (A) The upper map schematically depicts the end of chromosome 2 including the integrated plasmid concatamer (blue box) in 3D7/pBKminC parasites. kahrp promoter sequences are depicted by thick black lines. The upsC 5′ UTR sequence is depicted in red. The grey circles and squares represent the telomeric tract and telomere-associated repeat elements (TAREs) 1–6, respectively. Arrowheads indicate ORFs. The gene accession number refers to the most telomere-proximal upsB var gene PF3D7_0200100. The lower map shows a zoom-in view of the integrated concatamer (blue box). Restriction sites used in Southern analysis are shown by vertical dashed arrows, and expected fragment lengths are indicated and colour-coded. The hdhfr probe used for hybridisation is shown below the hdhfr-gfp coding sequence (grey box). (B) The autoradiograph shows the hybridisation results obtained with the hdhfr probe after digesting 3D7/pBKminC gDNA from unselected (−WR) and selected (+WR) populations with EcoRV/NcoI (red), EcoRV/SpeI (blue) or EcoRV/StuI (green). Note the presence of an additional hdhfr-containing fragment after each double-digest specifically in WR-selected, but not in unselected parasites (highlighted by purple arrows). In each case, the size of the additional fragment (schematically depicted to the bottom right) is approximately 2 kb smaller than the size of the EcoRV/NcoI, EcoRV/SpeI or EcoRV/StuI plasmid fragments (depicted to the top right). This result suggested the presence of a novel EcoRV site upstream of a single copy of hdhfr-gfp (highlighted in purple). i, integration event; p, plasmid fragment. (C) Ligation-mediated PCR. gDNA from WR-selected 3D7/pBKminC parasites was digested with EcoRV and NcoI and ligated into EcoRV/NcoI-digested pET-41 (EMD Biosciences). To amplify EcoRV/NcoI restriction fragments containing the hdhfr coding sequence, a primary PCR reaction was performed using T [file pone.0100183.s001.tif]

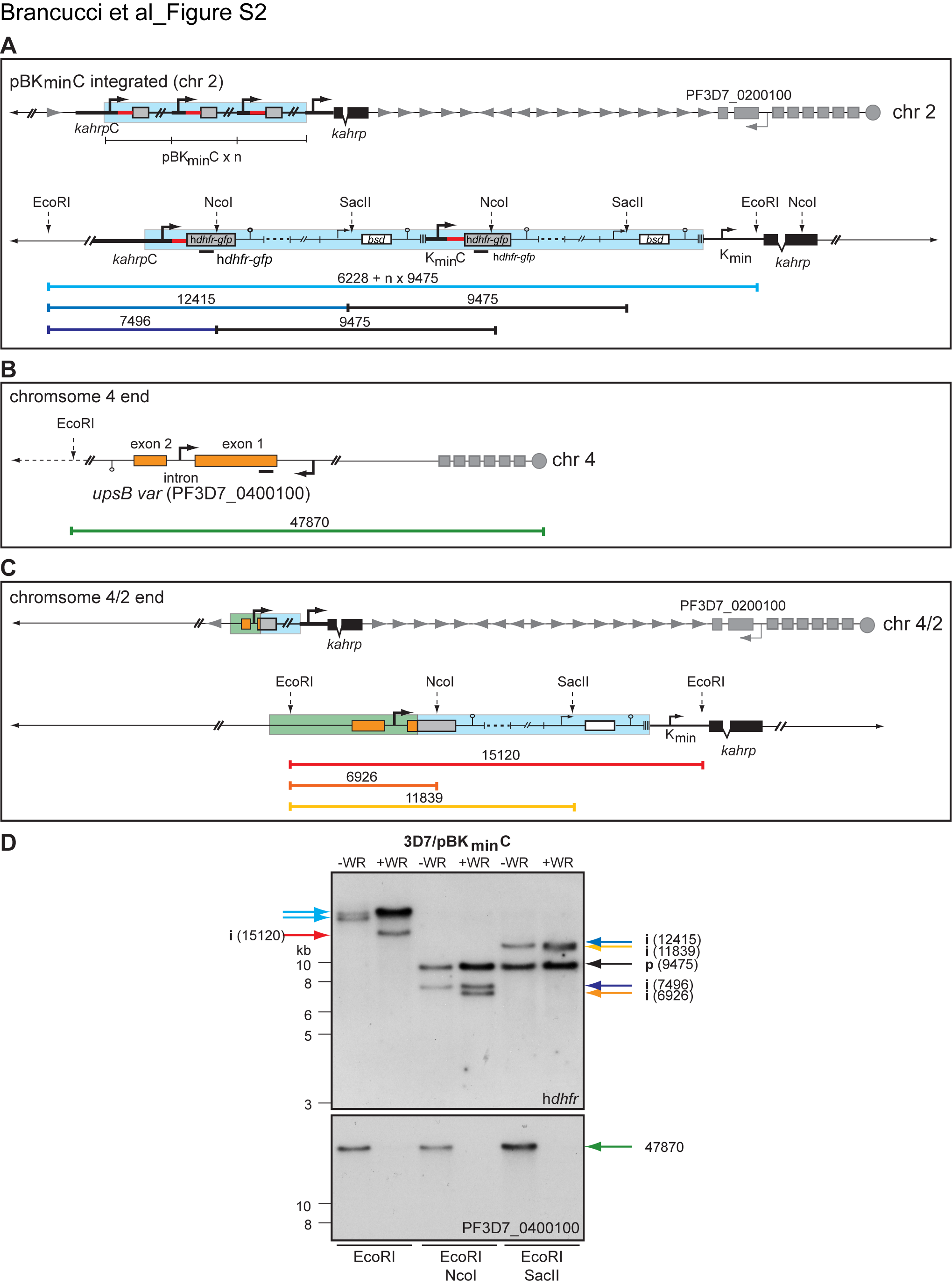

Supplement: Figure S2 — Further verification of the gene conversion event between chromosomes 2 and 4 in WR-selected 3D7/pBKminC parasites. (A) The map schematically depicts the end of chromosome 2 including the integrated plasmid concatamer (blue box) in 3D7/pBKminC parasites. kahrp promoter sequences are depicted by thick black lines. The upsC 5′ UTR sequence is depicted in red. The grey circles and squares represent the telomeric tract and TAREs 1–6, respectively. Arrowheads indicate ORFs. The gene accession number refers to the most telomere-proximal upsB var gene PF3D7_0200100. The lower map shows a zoom-in view of the integrated concatamer (blue box). Restriction sites used in Southern analysis are shown by vertical dashed arrows, and expected fragment lengths are indicated and colour-coded. The hdhfr probe used for hybridisation is shown below the hdhfr-gfp coding sequence (grey box). EcoRI sites are absent from the plasmid sequence. Hence, the EcoRI sites up- and downstream of the integrated concatamer release a restriction fragment in the size of 6228 bps (chromosomal DNA) plus n times 9475 bps (entire plasmid length) according to the number of copies in the concatamer. (B) The map schematically depicts the end of wild-type chromosome 4 including var gene PF3D7_0400100 (orange box) in unselected 3D7/pBKminC parasites. The PF3D7_0400100 exon 1 probe used for hybridisation is shown below the coding sequence. The position of the EcoRI restriction site downstream of the var locus and the expected fragment length are indicated. (C) The map schematically depicts the end of chromosome 4 after the gene conversion event between chromosomes 2 and 4 in WR-selected 3D7/pBKminC parasites (“chromosome 4/2 end”). The border between the green and blue boxes identifies the site of single-crossover recombination. The green and blue boxes represent sequences of the acceptor (chromosome 4) and donor (chromosome 2), respectively, of the gene conversion event. Restriction sites used in Southern analysi [file pone.0100183.s002.tif]

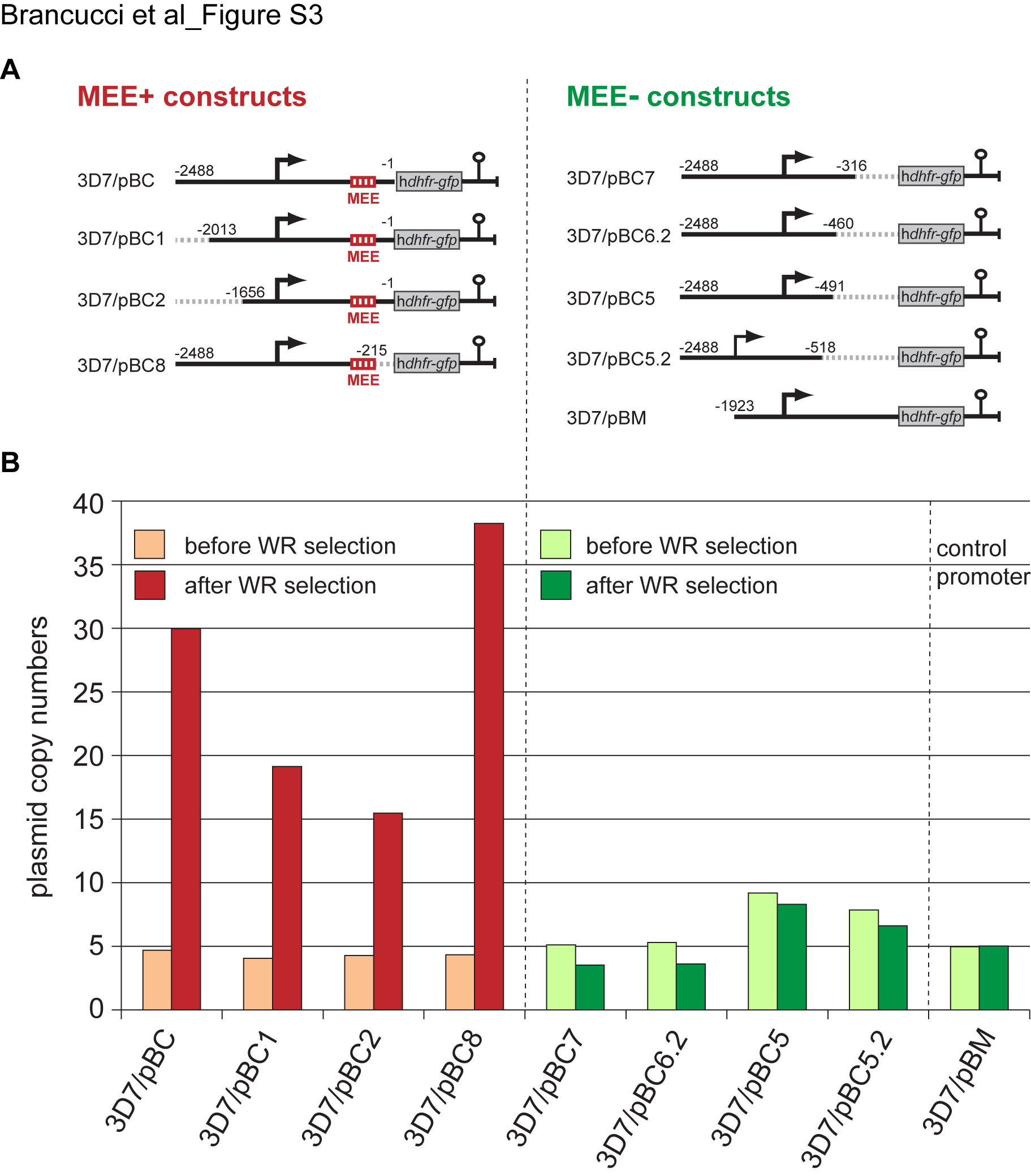

Supplement: Figure S3 — Plasmid copy numbers before and after WR selection. (A) Schematic depiction of upsC constructs that either retain the MEE (left panel; red) or lack the MEE (right panel; green) in the upstream sequence. Control plasmid pBM carries the mahrp1 promoter that naturally lacks a MEE element. (B) Average plasmid copy numbers before WR selection (light colours) and after WR selection (dark colours) in parasites transfected with MEE-positive constructs (red) or MEE-negative constructs (green). Plasmid copy numbers have been determined by qPCR and were calculated by dividing the absolute hdhfr-gfp copy numbers by the values obtained for the single copy gene msp8. (TIF) [file pone.0100183.s003.tif]
