# Supplementary material for: A var Gene Upstream Element Controls Protein Synthesis at the Level of Translation Initiation in Plasmodium falciparum
Source: PLoS One. 2014 Jun 17;9(6):e100183. doi: 10.1371/journal.pone.0100183 (PMC4061111; doi:10.1371/journal.pone.0100183)
Supplement: Table S1 — Primers used in this study. (PDF) [file pone.0100183.s004.pdf]

Table S1 Primers used in this study

	primer sequence (5`→3`)	RE sites	target gene
**pBKminC**			
kahrp-F-B	cagt**agatct**ctatgttagttatgataggacc	BglII	PF3D7_0202000
kahrp-R-N-S-B	cagt**gcggccgc**agt**cctgcagg**agt**ggatcc**atttttcaatcttttttttcagc	NotI, SbfI, BamHI	PF3D7_0202000
upsC-F-B	cagt**ggatcc**ccccatcacatatagtaggac	BamHI	PF3D7_1240600
upsC-R	cagt**gcggccgc**ttttgtttttttgtttatcgttcg	NotI	PF3D7_1240600
**upsC deletion constructs**			
pBC5.2-R-N	gatc**gcggccgc**ggggggaagaaaacgc	NotI	PF3D7_1240600
pBC6.2-R-N	gatc**gcggccgc**tggtagtcactatgtttg	NotI	PF3D7_1240600
**qPCR**			
PF13_0170F	tggctaggatatgattggaaagaaca		PF3D7_1331700
PF13_0170R	tacggttctatttctatatggtgaatca		PF3D7_1331700
gfpF	acacttgtcactactttcgcgtatggtcttc		
gfpR	accttcaaacttgacttcagcacgtgtcttgtagt		
kahrpF	acggatccggtgactccttcgat		PF3D7_0202000
kahrpR	tggtgaacctgtggtgcttggtgat		PF3D7_0202000
msp8F	tgacgcaaaagcaagggacaacaataataatgatga		PF3D7_0502400
msp8R	tcatcgtcatcatcattatcatcatcatcatcacc		PF3D7_0502400
**hybridisation probes**			
kahrpF	acggatccggtgactccttcgat		PF3D7_0202000
kahrpR	tggtgaacctgtggtgcttggtgat		PF3D7_0202000
hdhfrF	agctggatccgcggccgcaaaacatgcatggttcgctaaactg		
hdhfrR	agctgtcgacagcagcatcattcttctcatatacttcaa		
hsp86F	gaattgattagtaatgctagtg		PF3D7_0708400
hsp86R	gtttcatccttagtaactgtg		PF3D7_0708400
PFD0005wF	ccaaaaacacatatacagaaacaca		PF3D7_0400100
PFD0005wR	caacgaatgcagtttagcgga		PF3D7_0400100
**Ligation-mediated PCR**			
T7 terminator	gctagttattgctcagcgg		
hdhfr_R1	agctgtgcagagcagcatcattcttctcatatacttcaa		
hdhfr_R2	ccttgtggaggttccttgag		
hdhfr_R3	ggtagtccccgttcttgcc		
